# Hospital Meetings, &C.

**Published:** 1898-06-18

**Authors:** 


### HOSPITAL MEETINGS, &C.

### WORKING MEN AND HOSPITALS.

A meeting of the Conference of Delegates of Working Men's Clubs on the question of the London Hospital was held at the office of the London Chamber of Commerce on Wednesday evening, June 8th, under the presidency of Mr. Harold Boulton, vice-president of the Federation of Working Men's Social Clubs. The subject arranged for discussion was "The Better Management and Organisation of the Hospital System generally through the Establishment of a Central Board or otherwise."

The Secretary (Mr. Douglas Eyre) announced that Mr. C. S. Loch, secretary of the Charity Organisation Society, would open the discussion, but Sir Henry Burdett and Sir William Broadbent, who had hoped to attend, were unfortunately unable to be present. Sir Henry Burdett had, however, embodied his views in a letter, which he (Mr. Eyre) proposed to read at the conclusion of Mr. Loch's address.

Mr. Loch, after sketching the origin of the hospital system, quoted statistics relating to the number of out-patients, and proceeded to remark that a very large number who attended might well belong to friendly societies, but it seemed to him the hospitals acted as a means of drawing people from these societies. Touching on the question as to whether the work done in the out-patient department is good charitable work, Mr. Loch said that if you are dealing with a stranger in a charitable matter you ought to know what his circumstances are. It was not a question of anti-mendicity. The question was whether or not the right thing was done by the person in distress, and he would argue that on the whole the right thing was not done. Then was the work good medical work? On this point doctors differed. Some of the hospitals limited the number of patients, at others enormous crowds were dealt with. He thought that the proper test would be to deal with the number that would be dealt with by the doctors if they were in private practice. If this were done and inquiry made there would be a considerable decrease in the numbers. A great argument for some kind of change was that by association reforms might become possible which were now out of the question. It might be asked where would a central body get its funds from? Well, in the first place, aid might be sought from the City Parochial Charities, and in the second money might be secured in the way of legacies and from similar sources. The money so raised could be used in the way of bonuses to the various hospitals. The Prince of Wale Fund had adopted that plan, and it was alleged that individual hospitals had not suffered in consequence. With regard to the question of municipalising the hospitals, Mr. Loch said he thought it was desirable they should go on as they were going on at present. The hospitals under the voluntary system were well managed from the point of view of the medical staff, and a great deal of service and of money freely given would be lost if they were to cease to become voluntary institutions. But a Central Board would be a great consultative and educational centre, it would enable all interested to know more about hospitals than they do now. He had heard a great deal said upon this question, but it seemed to him that the main argument had not been touched, that a powerful charitable force would be created, which instead of pauperising men, in a manner, would strengthen the friendly societies and make medical relief serve more and more towards the independence of the people.

Mr. Eyre now read the following letter from Sir Henry Burdett:—

"Dear Sir,—I had hoped to be present, because I wished both sides to be heard, and none but accurate statements to be allowed to pass. As matters stand at present a great deal of misapprehension has arisen as to the management and organisation of the metropolitan hospitals, which at the present time are wonderfully efficient and admirable. The reason for this efficiency lies mainly in the circumstance that the hospitals are managed by gentlemen who give a large amount of time to the business, and who are whole-heartedly devoted to the work.

"I am against the setting up of a so-called Central Board, or otherwise, which is to consist of between 100 and 200 individuals, very few, if any, of whom have any special or practical knowledge of hospitals. Without knowledge and practical experience evil rather than good must result from the establishment of such a Board as has heretofore been advocated by the Charity Organisation Society and by a number of general practitioners of medicine who are discontented with the present state of things. As to the latter the public must one day realise what is the precise truth, that the remedy for any abuse of the hospitals, so far as it exists, lies to a large extent in the hands of the medical men themselves. There have been innumerable commissions and committees of inquiry on this subject. Many associations have been formed by medical men and others to promote reform, numerous conferences have been held, but the results directly due to such movements are practically nil. As a medical writer forcibly puts it, 'Notwithstanding all the talk of all these years, and notwithstanding the fact that the medical profession is an absolutely close corporation, and, moreover, that it possesses a powerful, wealthy, and representative association, which includes in its membership the staff of practically every hospital in the kingdom. Notwithstanding all this, things of which the medical profession is said to complain in regard to hospital abuse continue to go on under its very nose and with the concurrence and assistance of its most noted leaders. We think the inference is fair, namely, that the complaints which are from time to time made by the doctors against the hospitals are not so real as some would have us believe, and that so far as the public, and especially the giving public, are concerned, they may be put on one side until the medical profession offers something like an authoritative pronouncement as to the proper treatment to be applied to them.' At any rate, until something of this kind is done the idea that medical men at large are opposed to hospitals must be taken as erroneous and must on no account be held as a valid excuse for withholding a subscription.

"I hope the working men who attend your conference will fully grasp the fact that there is now a Central Hospital Council for London, originating with the supporters of the great metropolitan hospitals which have medical schools attached to them, of which many of the leading physicians, surgeons, and metropolitan hospital managers are members. It has already done some most useful work and is full of promise for the future.

"In addition to this H.R.H. the Prince of Wales has appointed a committee of representative men, presided over by the treasurer of St. Bartholomew's Hospital, to make an inspection of every London hospital which seeks a grant from the Prince of Wales's Fund. These two facts show that all the work and more than the work which the proposed Central Board could have attempted will now be done by practical men in a practical way, to the no small benefit of the public, the medical profession, and the patients, which last are after all the most important factors in the problem.

"The proposal to establish another body to be called the Central Board, or otherwise, which has been weighed in the balance of public and hospital opinion during the last six or seven years and been found wanting, is therefore a plan which in existing circumstances cannot be entertained or supported by thoughtful people who are possessed of that sound knowledge which comes of observation.—Believe me, yours faithfully, "Henry C. Burdett."

Mr. Eyre next read a letter that had appeared in the *Times*, in which Sir William Broadbent advocated the establishment of a "hospital assembly," elected by the hospitals and kindred institutions and "representative of all the interests concerned."

The Chairman then announced that Mr. Loch was prepared to answer any questions that might be put to him, whereupon

A Delegate inquired how, if it were not proposed to amalgamate the funds of the hospitals, could a Central Board call upon a poor hospital which had not money to carry out certain things the Board considered desirable?

To this Mr. Loch replied that what was proposed was that the Central Board should obtain funds from people who desired to help the hospitals generally, and not any hospital in particular. Those funds would be used to assist the hospitals to do what was wanted.

Another Delegate asked whether it was not the fact that the Prince of Wales's Fund did what Mr. Loch proposed?

The latter gentleman answered that Sir Henry Burdett's view was that it did. But the question of finance was not the whole question, and he believed that matters could be settled more satisfactorily by a self-governing body than by one which did not possess that advantage.

Cannot the hospitals take the question of inquiry into the position of out-patients in hand without a Central Board? demanded another Delegate.

Mr. Loch explained that the Central Board was suggested rather as an adjuster of hospitals—between one institution and another. The Hospital Sunday Fund said there ought to be an inquiry officer at each hospital, but what could the inquiry be where there were 150,000 or 87,000 patients in a year? What was wanted was co-operation. Still another delegate suggested that the number of out-patients was largely increased by surgical cases, with which the dispensary doctor would not deal, while in-patients who were requested to attend after they had been discharged also swelled the ranks of the out-patients. In reply to a further question, Mr. Loch, referring to Sir Henry Burdett's letter, asked, why should representatives necessarily be inefficient? He also protested that general practitioners were not opposed to hospitals, and statements to the contrary were absolutely misleading, and gave a wrong turn to the whole reform aimed at.

Would the Workmen's Club and Institute Union be represented on the Central Board? was the next question, and Mr. Loch replied that it was the idea of the proposers of the scheme that such bodies should have representation.

A Delegate: If they did you would have men who know nothing about hospitals. That is just what Sir Henry Burdett objects to.

To this Mr. Loch replied that in his opinion workmen would elect good representatives, and there were other interests besides those of the hospitals to be dealt with. It was not necessary that the representatives should know all about hospital work.

Questions having been brought to a close, a general desire was expressed to hold further meetings, and it was suggested that a representative of the Hospital Fund should be invited to attend. The name of Sir Sydney Waterlow was mentioned, and Mr. Loch suggested that of Dr. Glover, but the final arrangements were left in the hands of the secretaries.

### GROSVENOR HOSPITAL.

In the fine new building facing on to the playing fields of the Westminster School, the annual meeting of the Grosvenor Hospital for Women and Children was held on Friday, June 10th, under the presidency of the Marquis of Lothian. Mr. A. J. Ram, chairman of the Committee of Management, who proposed the adoption of the report and accounts, pressed upon the meeting the desirability of at once raising funds to build a new wing on the adjacent ground belonging to the hospital in order that they might be able to take children as in-patients, and not only as out-patients as at present. As an alternative, the idea of devoting one of the existing wards to children was mooted, but Dr. A. C. Butler-Smythe expressed his belief that the hospital was not fit to receive children—it was undoubtedly a new wing that was wanted, not a children's ward. Everything was ready—the architect, the builder, and even the plans; all that was lacking was the necessary money, and this it was hoped would be forthcoming now that the need for it was known. Mr. Ram's announcement that, thanks largely to the munificence of a lady, they were in possession of their new building free of debt was received with gratification by the meeting. But in common with the Chairman, Sir Charles Fremantle, Lord Charles Scott, and other speakers, Mr. Ram emphasised the paragraph in the report which pointed out that enlarged buildings and increased efficiency involve increased outlay, and that is now more than ever essential that the permanent income should be increased. A friend had offered £50 towards wiping off the existing overdraft of £500 if nine other sums of £50 were given within six months. It was announced that already six such additional sums had been promised, and it was hoped that the remainder would be forthcoming immediately. From the report it appeared that the income for 1897 amounted to £1,160, and the ordinary expenditure to £1,308, a deficiency on the year's work of £148. The number of in-patients during the year was only 91, owing to the fact that for the first ten months the temporary premises alone were available, the accommodation being restricted to five beds. The out-patients numbered 2,617, the attendances being 10,938.

### HOSPITAL FOR EPILEPSY AND PARALYSIS.

Canon Duckworth presided over the annual meeting of the Hospital for Epilepsy and Paralysis and other Diseases of the Nervous System, held on Monday last. The report stated that a new hospital building is now an imperative and urgent necessity, since the trustees of the property on which the hospital stands have decided not to renew the lease, which has now less than two years to run. The size of the future hospital must depend upon the amount placed at the committee's disposal, but £15,000 to £20,000 must, it is stated, sooner or later be raised. Up to the present the committee are able to count upon a sum of over £2,000, and the offer of Mr. Compton of £500 if nine other donations of similar amount be tendered is still open. The Prince of Wales's Fund is referred to at some length in the report, which asserts that as regards most hospitals, "The result is merely a repetition of the Hospital Sunday grant, being in the case of this hospital the sum of £57 10s , a very welcome addition to the income of the year, but scarcely commemorative of the great occasion which inspired His Royal Highness's benevolent movement." Reference is also made to the Central Hospital Council, and the committee add, "The finance of all hospitals receiving awards from the Hospital Sunday and Hospital Saturday Funds already come under the purview of the distribution committees of those bodies, and the question of a similar and independent examination on the part of the Council of the Prince of Wales's Hospital Fund has already been publicly referred to as possible, so that bodies of a more or less disciplinarian and critical character, whether really needed or not, will not apparently be wanting, at any rate, in number." According to the accounts, the total receipts for the year, including £220 brought forward, and £200 realised by tho sale of stock, amounted to £3,128. The total expenditure, including £1,000 transferred to the new building fund, was £2,932, but against the balance of £195 in hand on December 31st, there were liabilities amounting to £191. In the in-patient department 101 cases were under treatment, while the out-patient attendances amounted to 11,928, an increase of 509 on the figures for 1896, and of 2,683 on the average for the five preceding years. In moving the adoption of the report, Canon Duckworth said that great disappointment had been caused by the failure of the legacy of £4,000 which they expected to receive under the will of Mrs. Armitage. All hope of obtaining that amount must now be abandoned, so that instead of going to the public with £6,000 in hand for the new building fund, they had only £2,000. The Chairman made a strong appeal to the charitable on behalf of the hospital, as also did Sir Owen Burne, who seconded the resolution. The adoption of the report was carried unanimously.

### THE BOSCOMBE HOSPITAL.

Dwellers in Bournemouth to-day remember the time when that rapidly growing town was a tiny village, and the nearest butcher lived at Poole. Nowadays Bournemouth is a large and flourishing place, and the sounds of building are more and more heard in the land. On the eastern side the development has been the greater, and Boscombe, now practically a town of itself, contains a large proportion of the poorer population of the whole place. For this district the Boscombe Hospital, in Shelley Road, with ten beds, has remained the only hospital accommodation, and the need for additional beds is being greatly felt.

Early last year a committee was formed to go thoroughly into the matter and consider how the needful enlargement could best be managed, with the result that it became evident that the remodelling of the present building was impracticable for many reasons. Then an opportunity occurred for securing a very good site for a new building at the rear of the existing hospital, and after much consideration the purchase was effected for the sum of £1,500. For the hospital building fund some £1,300 was already in hand at the beginning of the year, but this could not be diverted towards the more immediate need, and a fund has, therefore, been started for the purpose of raising the needed purchase money.

The idea, therefore, is to leave the present hospital as it is, continuing its work without interruption, and upon the new site to erect a new building, in sections, as funds will permit. A commencement would be made by at once building two pavilions on the new site, one for men and one for women patients, connecting them with the present building by a temporary corridor. Then should follow with as little delay as may be a third ward for children. The new hospital will be built upon the pavilion principle, as being the one most hygienically perfect, for, as the committee remark in their report, "it is now recognised that sunshine is Nature's constant disinfectant, and that pure air is our best restorative to health, and the new scheme has been planned to allow the sun to shine into the wards the whole of the day, and to obtain perfectly free ventilation without draught."

And now to the moral of the tale. Here is a well managed little hospital doing excellent work, with its hands tied and its energies crippled for want of room, or, in other words, for want of money to start itself on larger lines. The following table will show what the increase in the work has been of late, while the number of beds has had to be decreased for hygienic reasons :—

| In-patients. | 1895. | 1896. | 1897. |
|---|---|---|---|
| Total number of admissions ... | 109 | 130 | 185 |
| Average number of days in hospital, per patient ... ... | 25·5 | 23·04 | 18·25 |
| Average number of beds occupied | 8·25 | 8·5 | 8·5 |
| Accidents ... ... ... ... | 11 | 18 | 40 |
| Operations ... ... ... ... | 63 | 100 | 121 |

In the out-patient department the increase is no less marked, the number of casualties treated having risen from 145 in 1895 to 401 last year. That a larger hospital is urgently wanted, and that the present one is and has been doing all it can to cope with the work before it are acknowledged facts. It only remains to find the money to carry out the new scheme as speedily as possible, and this surely ought not to be very difficult. The sum needed for the whole thing is not such a great matter, and Bournemouth contains many hundreds of people more or less rich in this world's goods to whom the needs of Boscombe should not plead in vain.

The hospital is fortunate in having such an hon. secretary as Mr. Evan F. Maberly, whose keen interest is shown by his unceasing energy on its behalf.

